# Using a Lethality Index to Assess Susceptibility of *Tribolium confusum* and *Oryzaephilus surinamensis* to Insecticides

**DOI:** 10.1371/journal.pone.0142044

**Published:** 2015-11-11

**Authors:** Paraskevi Agrafioti, Christos G. Athanassiou, Thomas N. Vassilakos, George Vlontzos, Frank H. Arthur

**Affiliations:** 1 Department of Agriculture Crop Production and Rural Environment, University of Thessaly, Volos, Greece; 2 USDA-ARS, Center for Grain and Animal Health Research, Manhattan, Kansas, United States of America; Australian National University, AUSTRALIA

## Abstract

We evaluated knockdown caused by four insecticides: alpha-cypermethrin, chlorfenapyr, pirimiphos-methyl and fipronil against adults of *Tribolium confusum* Jacquelin Duval, the confused flour beetle and *Oryzaephilus surinamensis* (L.), the sawtoothed grain beetle. Bioassays were conducted on concrete and metal surfaces. Adults of the tested species were exposed on both surfaces treated with the above insecticides at two doses (low and high). Knockdown assessment was done after 15, 30 and 60 min of adult exposure in the treated surfaces. Also, after 1, 3, 5, 7 and 14 d of exposure, a lethality index was calculated with an equation resulting to values from 0 to 100, where 100 indicated complete mortality and 0 complete survival. We also developed a lethality index by ranking each adult on each surface from 0 to 4, 0: adults moved normally, 1: adults were knocked down, but were able to walk for short intervals, 2: adults were knocked down and unable to walk, but with visible movement of antennae etc., 3: adults were knocked down, with very minimal movement of the tarsi and the antennae and 4: adults were dead (no movement). Knockdown of adults immediately after exposure (15–60 min) was higher for pirimiphos-methyl followed by alpha-cypermethrin, for both dose rates tested and species, but only on the metal surface. The lethality index was nearly 100 for all insecticides after 5d of exposure for *O*. *surinamensis*, while for *T*. *confusum* the adult lethality index was considerably lower for alpha-cypermethrin, suggesting that that recovery from knockdown occurred. Chlorfenapyr was the only insecticide that was more effective on concrete than on metal, while the reverse was noted for the other three insecticides. These results show that knockdown has different levels, which can be used as indicators of insect mortality or recovery.

## Introduction

Most of the contact insecticides that are currently being used to control stored product insect species are neurotoxic and their activity on adults is directly related to specific changes in their behavior, which can occur during or after exposure to the treated substrate. In this context, the most important behavioral change is the immobilization of the exposed individuals, broadly described as “knockdown”. This can be defined as inability of adults to walk in an upright manner “paralysis, whether reversible or not” [1], or “moribund” [2], a state in which the adults are alive but exhibit only reflex twitching in extremities. Nevertheless, most of the studies available on the efficacy of contact insecticides, which are applied either directly on grains or as surface or crack and crevice treatments, are focused on the evaluation of mortality *per se* without taking into account the knockdown patterns, or how knockdown is eventually related to mortality.

In a recent study, Tsaganou et al. [3] conducted studies with the neonicotinoid thiamethoxam, a neurotoxic insecticide, and reported that high knockdown of adults of the lesser grain borer, *Rhyzopertha dominica* (F.) (Coleoptera: Bostrychidae), the confused flour beetle, *Tribolium confusum* Jacquelin du Val (Coleoptera: Tenebrionidae) and the sawtoothed grain beetle, *Oryzaephilus surinamensis* (L.) (Coleoptera: Silvanidae) exposed on treated grains, and that, in general, knockdown was positively associated with mortality. However, the authors noted that seven days after the removal of the exposed individuals from the treated grains, adults began to recover from knockdown. Similarly, Arthur [4] conducted studies by exposing adults of *R*. *dominica* on wheat treated with a mixture of chlorpyriphos-methyl and deltamethrin, and noted increased survival and recovery after the removal of the exposed adults from the treated commodity. Conversely, Athanassiou et al. [5] reported delayed mortality of adults of the same species that initially survived exposure on treated grains, and one week later were dead after they were transferred to untreated grains. These results describe the phenomenon of “delayed mortality”, which in many cases is expressed more vigorously than “initial mortality”. The interval between initial and delayed mortality can include different levels of continuous or interrupted knockdown, especially in exposure studies with neurotoxic insecticides. Paradoxically, the connection between knockdown and mortality is poorly understood and it is generally uncertain if knockdown is a reliable indicator of the concomitant mortality.

There are insecticides that are currently available for use in stored product protection that are not neurotoxic, and therefore exposure may not produce the behavioral changes associated with knockdown. One example is insect growth regulators (IGRs) which affect molting and development of immatures but do not affect adults. Knockdown as a behavioral state is somewhere on the continuum between survival, which is often equated with normal movement, and mortality, where the insect cannot move even when touched or prodded. Some exposure studies merge knockdown and mortality into one value and classify insects as "affected" and assess recovery [6]. However, it is apparent that there are different levels or degrees of knockdown, ranging from interrupted walking to minimal movement (i.e. in tarsi or antennae). Consequently, the “degree of knockdown”, e.g. “strong” or “weak” knockdown could be evaluated to determine if would be an accurate predictor of recovery or eventual death.

Nevertheless, there is still inadequate information of the quantitative assessment of the plasticity of knockdown and an index of lethality. Athanassiou et al. [7], reported a rapid knockdown of adults of *T*. *confusum* and the red flour beetle, *Tribolium castaneum* (Herbst) (Coleoptera: Tenebrionidae) for a combination of the pyrethroid beta-cyfluthrin with the neonicotinoid imidacloprid on concrete, but mortality after 7 d of exposure remained at relatively low levels. Still, there are studies that indicate that the application of neurotoxic insecticides may increase mobility [8–10].

Leksey et al. [2] conducted studies with the brown marmorated stink bug, *Halyomorpha halys* (Stal) (Hemiptera: Pentatomidae), and showed increased knockdown was positively correlated with mortality after exposure to insecticides. In that study, the authors developed a “lethality index”, which was based on the proportion of individuals that were dead, knocked down (moribund) and alive. This index ranged between 0 and 100, indicating complete survival or complete mortality, respectively. In the calculation of this index, knockdown was regarded as a uniform condition between mortality and survival, without taking into account the different levels of the moribund state. There are no studies that have differentiated knockdown in stored product insects exposed to an insecticide and related the levels of knockdown to eventual mortality. The objective of this study was to develop an improved knockdown index, expanding on the approach presented by Leskey et al. [2]. We used two major stored product insect species, *T*. *confusum* and *O*. *surinamensis*, and several insecticides with different modes of action. These insecticides were applied on two different surfaces, concrete and galvanized steel.

## Materials and Methods

### Test insects and insecticides

The test insects were obtained from field strains originally collected in 2005 and maintained at the Laboratory of Agricultural Zoology and Entomology, Faculty of Plant Science, Agricultural University of Athens, Greece. These strains were transferred to the University of Thessaly in Volols, Greece in 2009. Adults of mixed age and sex were used in the tests. The insects were reared in incubator chambers at 25°C, 65% Relative Humidity (RH) and in continuous darkness. *Tribolium confusum* was reared on wheat flour and *O*. *surinamensis* on oat flakes.

The insecticides used were: alpha-cypermethrin (5.8% w/w Active Ingredient [AI], Fendona 6SC, BASF Hellas) at 0.0015 mg active ingredient [AI] / cm^2^ and 0.003 mg AI / cm^2^, chlorfenapyr (21.45% w/w [AI], Phantom SC, BASFcorporation U.S.A) at 0.055 mg AI / cm^2^ and 0.11 mg AI / cm^2^, pirimiphos-methyl (49% w/w [AI], Actellic 50EC, Syngenta Crop Protection AG (Basel, Switzerland)) at 0.05 mg AI / cm^2^ and 0.1 mg AI / cm^2^ and fipronil (9.1% w/w [AI], Termidor 9SC, BASF Hellas) at 0.001 mg AI / cm^2^ and 0.002 AI / cm^2^. The doses used were based on the label dose of the above insecticides for application on surfaces. All insecticides were applied as water-based solution. The preparation of the solutions to obtain the above-mentioned concentrations, respectively, was made by diluting the appropriate amount of insecticide formulation in distilled water in 25 ml volumetric flasks to correspond with label directions.

### Surface treatments and bioassays

Petri dishes (90 mm in diameter, 15 mm high, 63.61 cm^2^) were used as the experimental units. Two types of surfaces that are commonly used in storage facilities were created in bottoms of the petri dishes, one with concrete and one with galvanized steel (metal), as described in detail by Vassilakos et al. [11]. Briefly, a water-cement mixture was prepared (Rockite^®^ for anchoring and patching, Hartline Products Co. Inc., Cleveland, OH, USA) by mixing 260 ml of water with 1 kg of powder, and the solution was poured into petri dish bottoms to create the concrete arenas. The metal surfaces were created in a local machinery shop using laser cutting equipment. After the metal disks were cut in round shape, they fitted and sealed into the dish bottoms, using hot glue (Bison Glue Gun Hobby, Bison International B.V., The Netherlands) to affix the metal disks to the Petri dishes. The internal walls of all the Petri dishes were treated with polytetrafluoroethylene dispersion (Sigma—Aldrich Co, Germany), to prevent insects from escaping. After the surface preparation, the insecticides were applied on the surfaces by using a Kyoto BD-183K airbrush (Grapho-tech, Japan). Spraying of each petri dish was done with 1 ml of the appropriate solution of dose rate. Additional series of dishes were sprayed with distilled water and served as controls. There were three dishes for each insect-insecticide-dose-surface combination (3 sub-replicates), and the entire procedure was repeated three times, by preparing new dishes each time (3 replicates).

### Knockdown and lethality

After spraying was completed, ten adults of each species were introduced into each dish (with different dishes for each species), plus 0.5 g of wheat flour as a food source, and placed at the conditions mentioned above. Then, knockdown was assessed after 15, 30 and 60 min. After the termination of this procedure, the condition of the exposed individuals was calculated with the use of a new Standardized Lethality Index, expanding on the index used by Leksey et al. [2], after 1, 3, 5, 7 and 14 d of exposure. Based on this index, each adult within the dish was ranked from 0 to 4, similar to the Likert-scale approach used to assess responses to questionnaires in that it measures the intensity of the response [12]. In our scale the responses were 0: adults moved normally, 1: adults were knocked down, but were able to walk for short intervals, 2: adults were knocked down and unable to walk, but with visible movement of antennae, legs, etc., 3: adults were knocked down, with very minimal movement of the tarsi and the antennae and 4: adults were dead (no movement). Thus, the lethality Index was calculated with the equation:

$$\sum_{0}^{4}\left(\frac{\left(N.al.\times w_{1}\right)+\left(N.1\times w_{2}\right)+\left(N.2\times w_{3}\right)+\left(N.3\times w_{4}\right)+\left(N.dead\times w_{i}\right)}{N.ad.\times N.ob\times {\text{max}}_{wi}}\right)\times 100$$

- Lethality coefficient *W*_1:_ 0.0, *W*_*2*_: 0.1, *W*_3:_ 0.2,*W*_4:_ 0.3, *W*_i:_ 0.4
- *N*.*al*: Number of adults alive
- *N1*: Number of adults at rank 1
- *N2*: Number of adults at rank 2
- *N3*: Number of adults at rank 3
- *N*.*dead*: Number of dead adults
- *N*. *ad*.: Total number of adults
- *N*. *ob*: Number of observations (one day, one observation)
- *max*w_j_: Maximum lethality coefficient

In order to determine the value of the weights w_j_, each one of the scale elements is divided by sum of the Likert scale. Therefore, for this specific research *w*_1_ = 1/(0+1+2+3+4) = 0.1, *w*_2_ = 2/(0+1+2+3+4) = 0.2, etc. The values of the index ranged between 0 and 100. Index scores close to 0 means that is very close to survival, or “weak” knockdown, while Index scores close to 100 mean mortality or “strong” knockdown.

### Data Analysis

Before the analysis, normal distribution of the data was tested by using the O'Brien test at 0.01 [13]. Knockdown assessments were analyzed, separately for each species, by using the MANOVA Fit Repeated Measures Procedure with Wilk’s lambda estimate, with JMP software version 7 [14], with dose rate, surface and insecticide as main effects, and time-knockdown as the repeated variable. Knockdown in the controls was not included in the analysis because there was no knockdown. The same procedure was also followed in the case of the Index, and means were separated by the Tukey-Kramer HSD test at 0.05 [15].

## Results

### Knockdown

**Fig 1:**
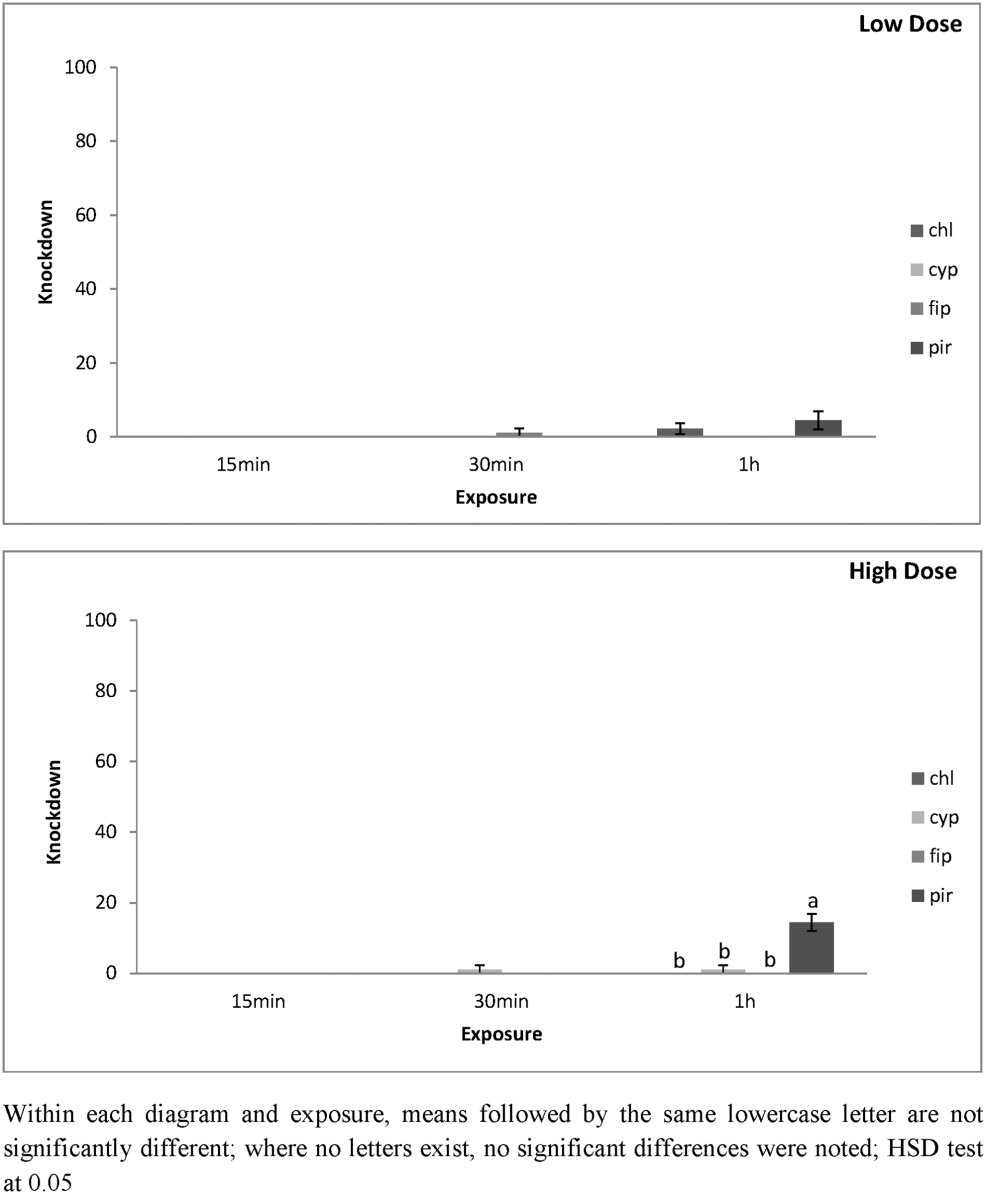
Mean Knockdown of *T*. *confusum* adults on concrete surfaces at low and high dose. Mean Knockdown (% ± SE) of *T*. *confusum* adults on concrete treated with chlorfenapyr (chl), alpha-cypermethrin (cyp), fipronil (fip) and pirimiphos-methyl (pir), at the low and the high dose rate of each insecticide after 15 min, 30 min and 1 h exposure intervals.

**Fig 2:**
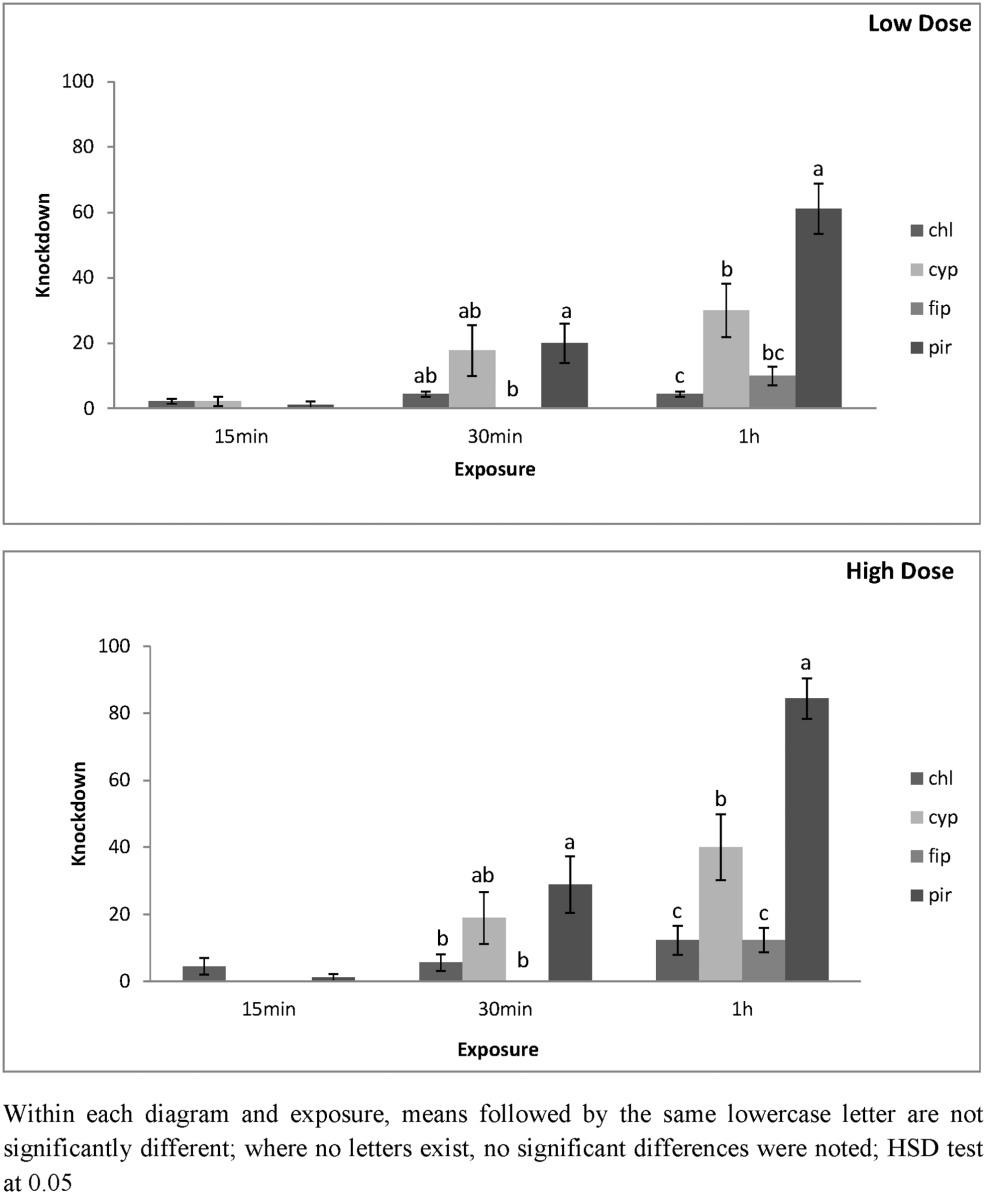
Mean Knockdown of *T*. *confusum* adults on metal surfaces at low and high dose. Mean Knockdown (% ± SE) of *T*. *confusum* adults on metal treated with chlorfenapyr (chl), alpha-cypermethrin (cyp), fipronil (fip) and pirimiphos-methyl (pir), at the low and the high dose rate of each insecticide after 15 min, 30 min and 1 h exposure intervals.

For both species, all main effects were significant, but some interactions were not (Table 1). The increase of exposure increased knockdown at different patterns between the two surfaces. For *T*. *confusum*, knockdown on concrete was generally low and did not exceed 20% for any of the combinations used (Fig 1). In contrast, for this species knockdown on metal was notably higher. The highest knockdown level was the pirimiphos-methyl treatment. Knockdown after 30 min at the lowest dose on metal, after 30 min was about 20%, but after 60 min knockdown exceeded 61% (Fig 2). Similarly, at the highest dose, the respective knockdown at 30 and 60 min. was 29 and 84% (Fig 2). For the other insecticides, knockdown was notably lower than that of pirimiphos-methyl and did not exceed 40%. The lowest knockdown for *T*. *confusum*, regardless of the surface and the exposure, was on the chlorfenapyr and fipronil treatments.

**Table 1:** Repeated-measures ANOVA parameters for *T*. *confusum* and *O*. *surinamensis* adult knockdown.

		*T*. *confusum*		*O*. *surinamensis*	
	dF	*F*	*P*	*F*	*P*
All Between	15	18.58	<0.01	51.95	<0.01
Intercept	1	146.80	<0.01	559.54	<0.01
Surface	1	111.93	<0.01	9.47	<0.01
Insecticide	3	30.88	<0.01	225.89	<0.01
SurfaceXInsecticide	3	21.15	<0.01	11.27	<0.01
Dose	1	3.96	0.05	18.13	<0.01
SurfaceXDose	1	2.05	0.15	0.93	0.34
InsecticideXDose	3	1.30	0.28	11.49	<0.01
SurfaceXInsecticideXDose	3	0.27	0.84	1.61	0.19
All Within Interactions	30	12.43	<0.01	14.35	<0.01
Time	2	141.83	<0.01	279.60	<0.01
TimeXSurface	2	92.90	<0.01	0.61	0.55
TimeXInsecticide	6	30.90	<0.01	63.91	<0.01
TimeXSurfaceXInsecticide	6	17.66	<0.01	3.17	<0.01
TimeXDose	2	6.46	<0.01	4.13	0.02
TimeXSurfaceXDose	2	2.40	0.09	1.04	0.35
TimeXInsecticideXDose	6	1.66	0.13	1.26	0.27
TimeXSurfaceXInsecticideXDose	6	0.33	0.92	0.43	0.86

**Fig 3:**
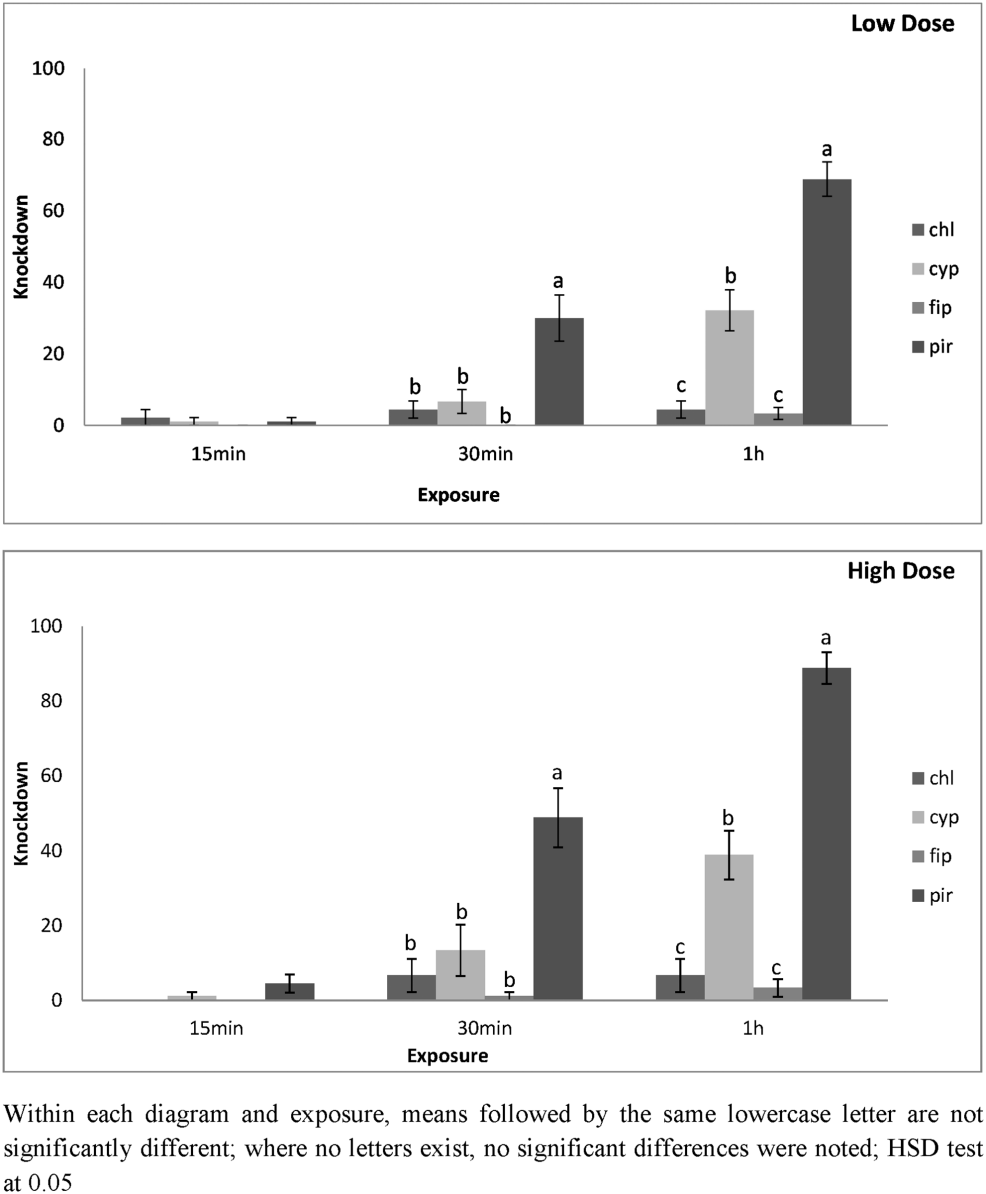
Mean Knockdown of *O*. *surinamensis* adults on concrete surfaces at low and high dose. Mean Knockdown (% ± SE) of *O*. *surinamensis* adults on concrete treated with chlorfenapyr (chl), alpha-cypermethrin (cyp), fipronil (fip) and pirimiphos-methyl (pir), at the low and the high dose rate of each insecticide after 15 min, 30 min and 1 h exposure intervals.

**Fig 4:**
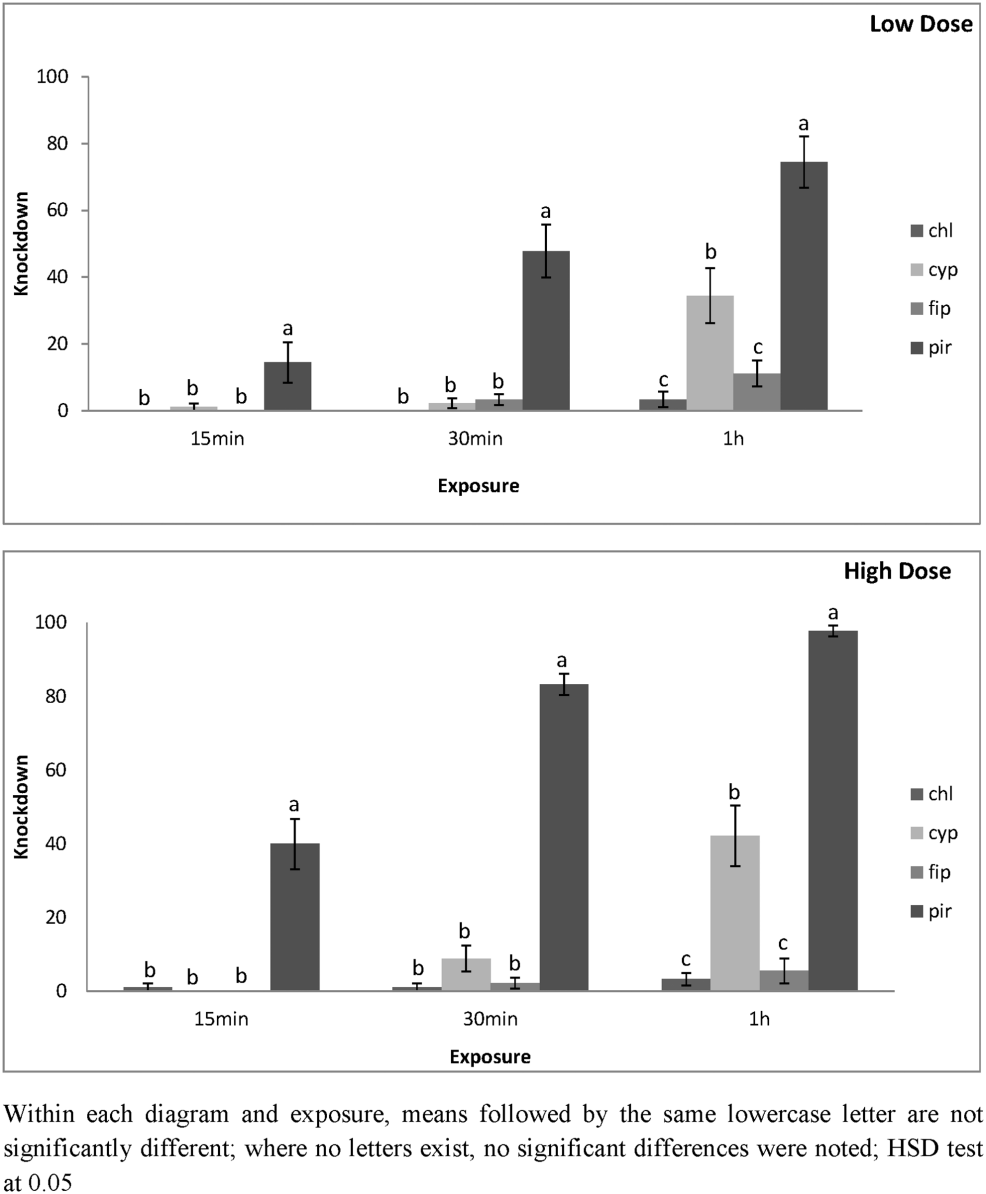
Mean Knockdown of *O*. *surinamensis* adults on metal surfaces at low and high dose. Mean Knockdown (% ± SE) of *O*. *surinamensis* adults on metal treated with chlorfenapyr (chl), alpha-cypermethrin (cyp), fipronil (fip) and pirimiphos-methyl (pir), at the low and the high dose rate of each insecticide after 15 min, 30 min and 1 h exposure intervals.

Similar results were also obtained for *O*. *surimamensis*. On concrete, knockdown was higher on pirimiphos-methyl, and after 30 min, was 30 and 49% for the lowest and highest dose, respectively (Fig 3). After 60 min, knockdown on concrete treated with pirimiphos-methyl was 69 and 89% for the lowest and highest dose, respectively (Fig 3). Conversely, for apha-cypermethrin, knockdown on concrete was 32 and 39% for the lowest and highest dose, respectively. Knockdown on concrete was negligible for the other two insecticides. For the lowest dose of pirimiphos-methyl, knockdown after 60 min of exposure was 74% (Fig 4). Almost all *O*. *surimamensis* were knocked down after 60 min of exposure to the highest dose. At the same conditions, knockdown for the lowest and the highest doses of alpha-cypermethrin was 34 and 42%, respectively. Even on metal knockdown was very low for chlorfenapyr and fipronil.

### Lethality Index

**Fig 5:**
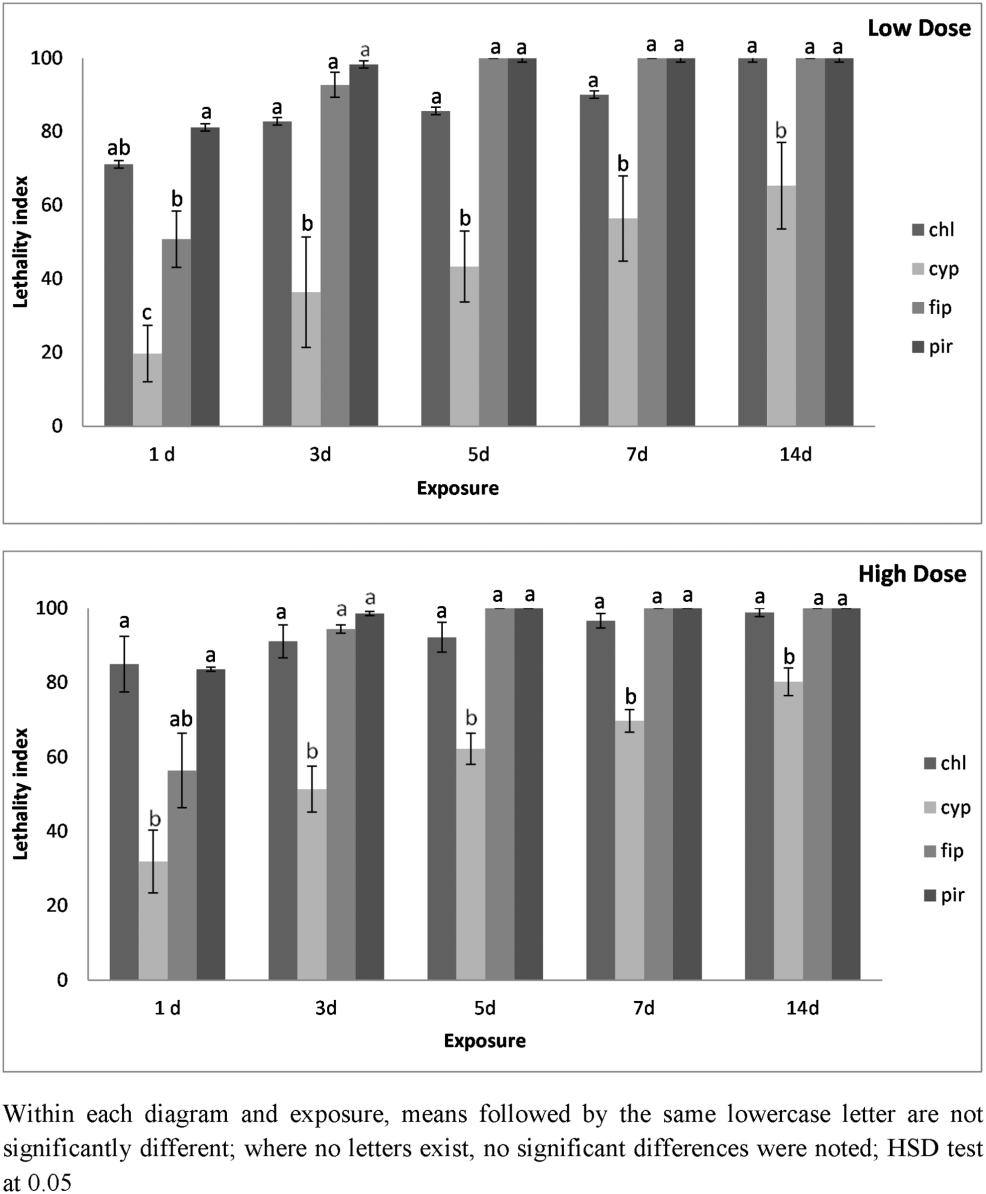
Lethality index of *T*. *confusum* adults on concrete surfaces at low and high dose. Mean lethality index (± SE) of *T*. *confusum* adults on concrete treated with chlorfenapyr (chl), alpha-cypermethrin (cyp), fipronil (fip) and pirimiphos-methyl (pir), at the low and the high dose rate of each insecticide after 1, 3, 5, 7 and 14 d of exposure.

For both species insecticide and surface x insecticide were significant, while surface was significant only for *T*. *confusum* (Table 2). In general, for all species-insecticide-dose-surface combinations, the increase in exposure, led to increased index values. For *T*. *confusum*, at the lowest dose of all insecticides on concrete, the lowest index values were recorded for alpha-cypermethrin, where the highest value after 14 d of exposure was 65 (Fig 5). In contrast, for fipronil and pirimiphos-methyl, the highest index value (100) was recorded from the 5^th^ day of exposure onward. Finally, for chlorfenapyr, the index reached 100 after 14 d of exposure. At the highest dose rate of all insecticides on concrete, the value of the index was only slightly increased for alpha-cypermethrin (Fig 5). The index reached its highest value for the other insecticides on the 5^th^ day of exposure, with the exception of chlorfenapyr.

**Table 2:** Repeated-measures ANOVA for *T*. *confusum* and *O*. *surinamensis* adult Lethality index (in all cases total error df = 32).

		*T*. *confusum*		*O*. *surinamensis*	
	dF	*F*	*P*	*F*	*P*
All Between	15	11.7	<0.01	7.6	<0.01
Intercept	1	6072.2	<0.01	23838.9	<0.01
Surface	1	9.8	<0.01	3.5	0.07
Insecticide	3	43.3	<0.01	27.9	<0.01
SurfaceXInsecticide	3	8.0	<0.01	7.0	<0.01
Dose	1	5.6	0.02	2.4	0.13
SurfaceXDose	1	0.1	0.73	0.7	0.42
InsecticideXDose	3	1.4	0.27	0.6	0.63
SurfaceXInsecticideXDose	3	0.4	0.72	0.3	0.85
All Within Interactions	60	2.9	<0.01	2.7	<0.01
Time	4	64.8	<0.01	53.9	<0.01
TimeXSurface	4	2.1	0.10	1.1	0.36
TimeXInsecticide	12	12.8	<0.01	10.0	<0.01
TimeXSurfaceXInsecticide	12	0.7	0.74	2.8	<0.01
TimeXDose	4	1.6	0.20	0.8	0.55
TimeXSurfaceXDose	4	0.4	0.81	0.5	0.72
TimeXInsecticideXDose	12	1.2	0.28	0.9	0.57
TimeXSurfaceXInsecticideXDose	12	0.3	0.99	0.6	0.82

**Fig 6:**
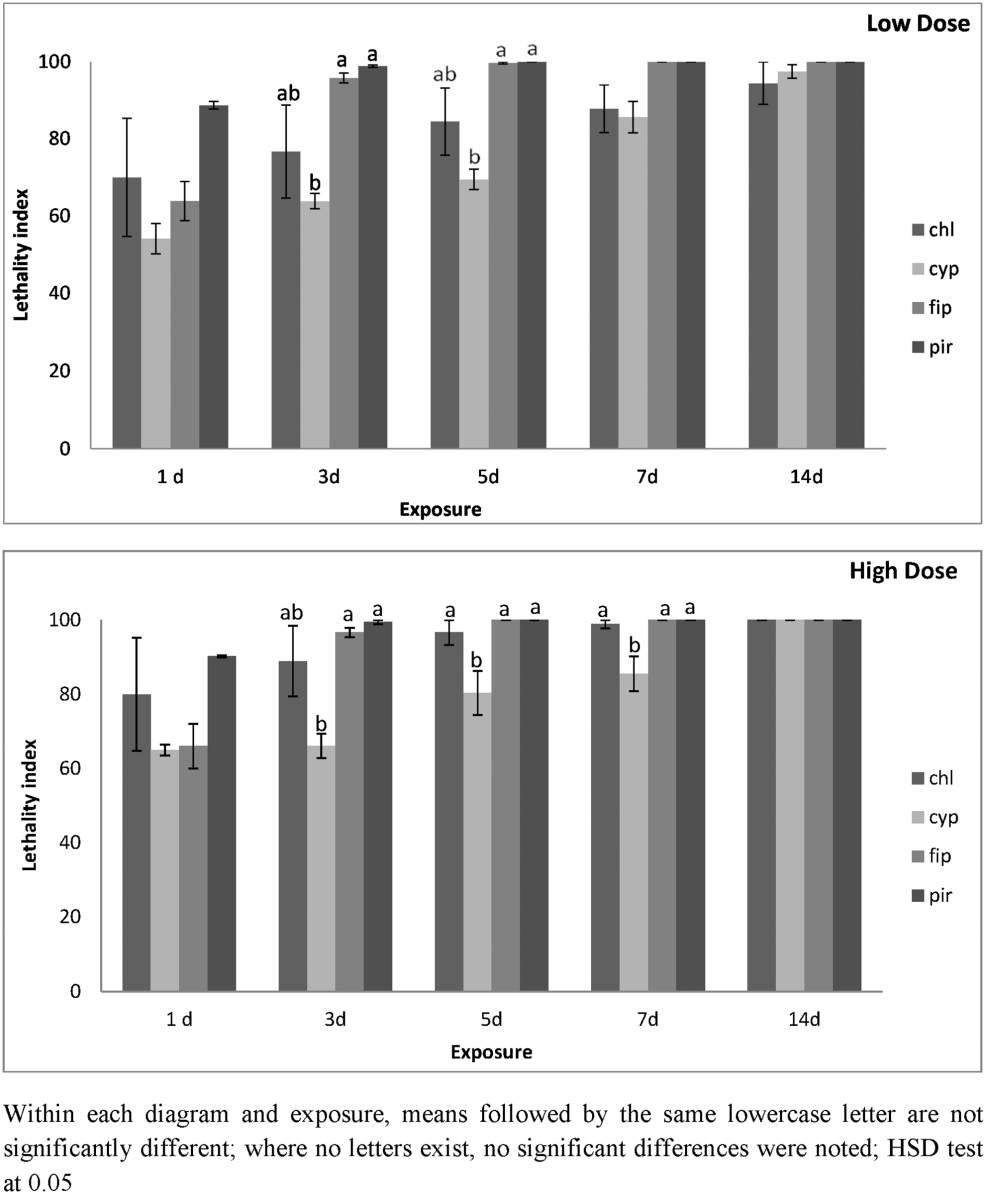
Lethality index of *T*. *confusum* adults on metal surfaces at low and high dose. Mean lethality index (± SE) of *T*. *confusum* adults on metal treated with chlorfenapyr (chl), alpha-cypermethrin (cyp), fipronil (fip) and pirimiphos-methyl (pir), at the low and the high dose rate of each insecticide after 1, 3, 5, 7 and 14 d of exposure.

For the same species on metal, in the case of alpha-cypermethrin, the index approached 100 only after 14 d of exposure (Fig 6). For pirimiphos-methyl and fipronil, the index approached 100 after the 3^rd^ day of exposure. For chlorfenapyr, the index did not reach the value of 100 even after 14 d of exposure. The increase of dose further increased mortality, and for firponil, pirimiphos-methyl and chlorfenapyr the index was at or near 100 at the 5-d exposure interval (Fig 6). In contrast, for alpha-cypermethrin, the index was 80 at this same interval, and reached 100 only after 14 d of exposure.

**Fig 7:**
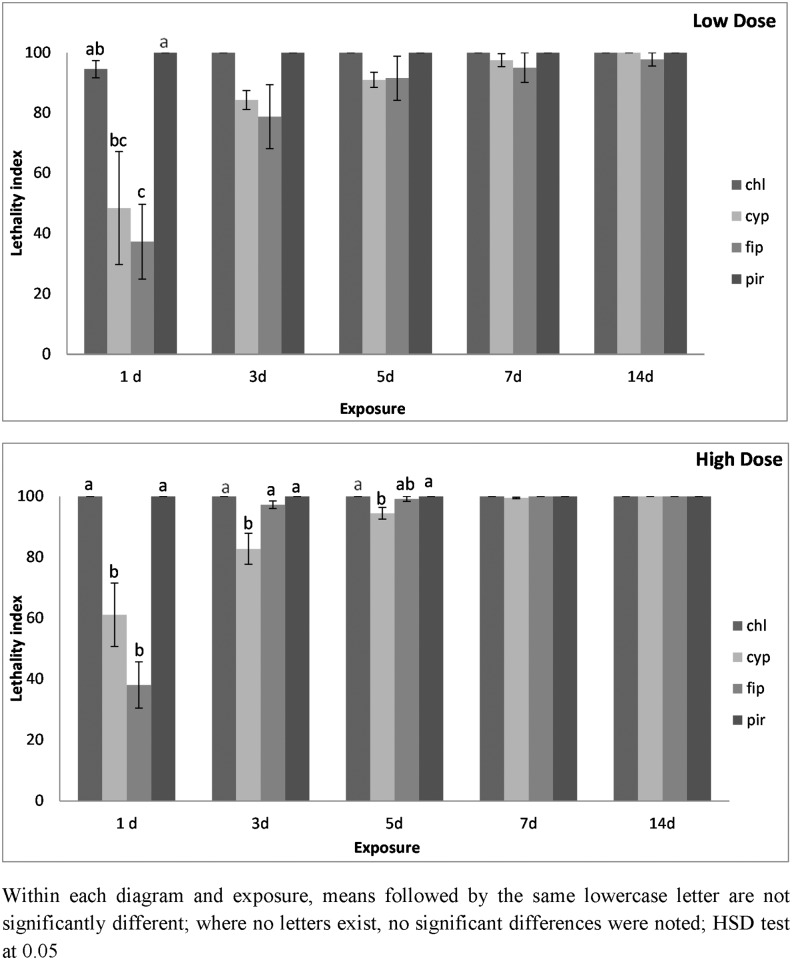
Lethality index of *O*. *surinamensis* adults on concrete surfaces at low and high dose. Mean lethality index (± SE) of *O*. *surinamensis* adults on concrete treated with chlorfenapyr (chl), alpha-cypermethrin (cyp), fipronil (fip) and pirimiphos-methyl (pir), at the low and the high dose rate of each insecticide after 1, 3, 5, 7 and 14 d of exposure.

The performance of the four insecticides was different for *O*. *surimamensis*. On concrete treated with the lowest dose of pirimiphos-methyl, the index value was 100 from the 1^st^ day of exposure (Fig 7). Similarly, for the lowest dose of chlorfenapyr, the index was 100 after 3 d of exposure. In contrast, for the other two insecticides, the index value was considerably lower, despite the fact that significant differences among insecticides were noted only at the 1^st^ day of exposure. At the highest dose on concrete, the index value was 100 for pirimiphos-methyl and chlorfenapyr from the 1^st^ day of exposure, while the index values for fipronil and alpha-cypermethrin were 38 and 61, respectively (Fig 7). Moreover, for alpha cypermethrin, the index value was significantly lower from that for pirimiphos-methyl and chlorfenapyr until the 5^th^ day of exposure.

**Fig 8:**
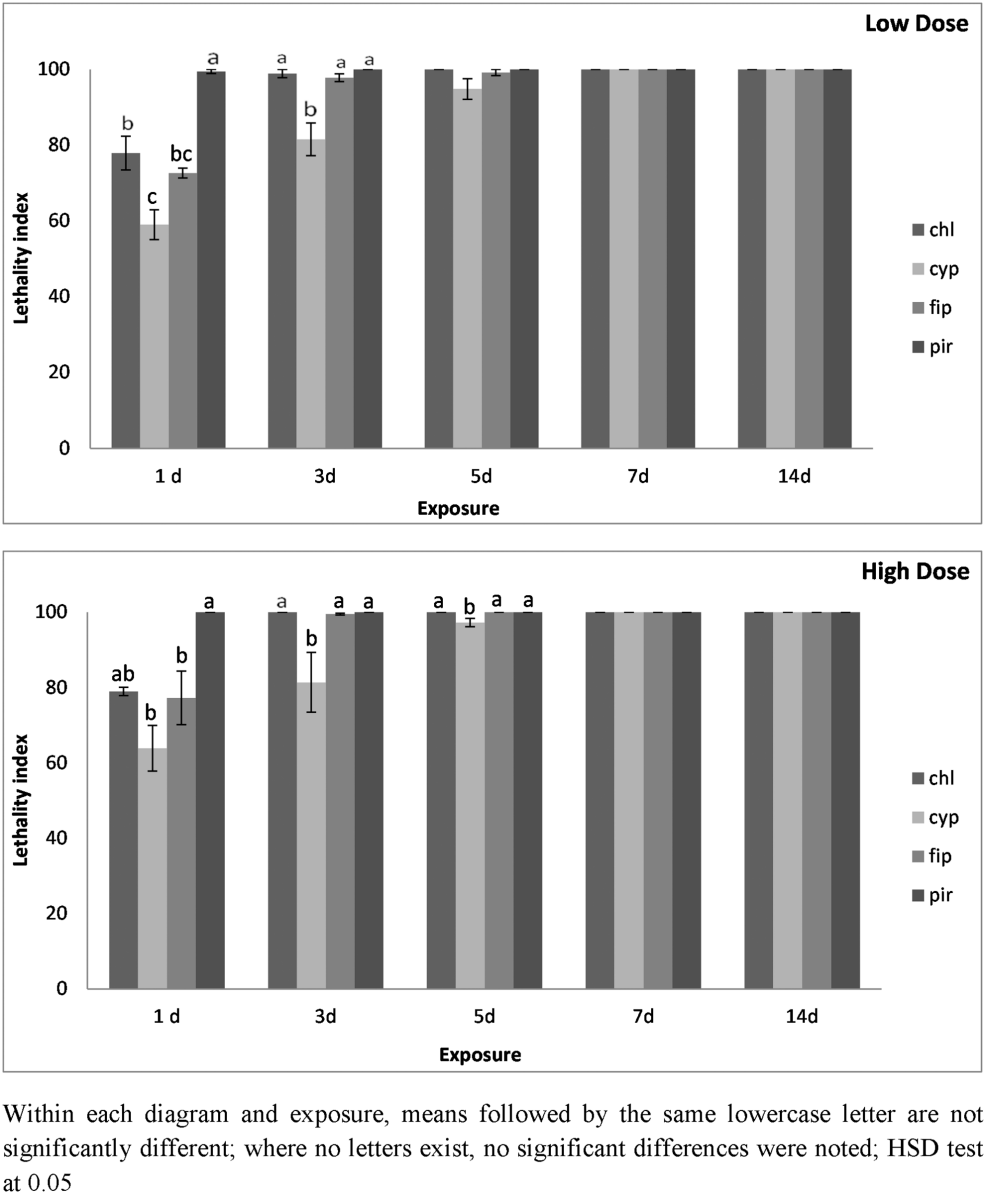
Lethality index of *O*. *surinamensis* adults on metal surfaces at low and high dose. Mean lethality index (± SE) of *O*. *surinamensis* adults on metal treated with chlorfenapyr (chl), alpha-cypermethrin (cyp), fipronil (fip) and pirimiphos-methyl (pir), at the low and the high dose rate of each insecticide after 1, 3, 5, 7 and 14 d of exposure.

On metal, for *O*. *surinamensis* adults, after 1 d of exposure at the lowest dose, the index value was 99.4 for pirimiphos-methyl, while for the other insecticides it did not exceed 78 (Fig 8). Nevertheless, no significant differences were noted among insecticides for the last three exposure intervals examined, while for all insecticides, the index value was 100 after 7 d of exposure. For the highest dose, the index was 100 for pirimiphos-methyl from the 1^st^ day and for chlorfenapyr from the 3^rd^ day, while for the other two insecticides, the index reached its maximum value on the 7^th^ day of exposure (Fig 8). The index for alpha-cypermethrin was significantly lower than that for the other three insecticides until the 7^th^ day of exposure.

## Discussion

In the present study, we evaluated four insecticides with different modes of action. Pirimiphos-methyl is an organophosphorus (OPs) compound that acts on the insects’ nervous system through acetylcholinesterase inhibition, while alpha-cypermethrin is a pyrethroid that acts neurotoxically through the sodium channel. Fipronil is also a neutotoxic insecticide, but with a completely different mode of action than OPs or pyrethroids. Fipronil is a pyrazole which acts as an antagonist to GABA (gamma-aminobutyric acid) receptors. Chlorfenapyr was selected for this study as it is a non-neurotoxic pyrrole that causes oxidative phosphorylation in the mitochondria, which disrupts the synthesis of ATP. Pirimiphos-methyl, alpha-cypermethrin and chlorfenapyr are widely used in several parts of the world in food processing facilities for surface and/or crack and crevice treatments [7, 16–17], while fipronil is used in the urban environment against ants, termites and cockroaches, as well as in veterinary medicine [18]. These differences in modes of action were selected in order to have a diverse range of behavioral changes of the adults of the two species examined, to differentiate the rank scaling of the index.

As expected, knockdown of adults immediately after exposure (15–60 min) was higher for pirimiphos-methyl and alpha-cypermethrin, for both dose rates tested, but only on metal. Knockdown was rapid especially for pirimiphos-methyl. To a lesser extent, immediate knockdown for alpha-cypermethrin was also higher than that for fipronil and chlorfenapyr on metal, but a considerable proportion of knocked down adults did not die during the first five days of the observation period. Sehgal et al. [19] found that knockdown of different stored product beetle species on concrete was higher with a mixture of the OP chlorpyriphos-methyl with the pyrethroid deltamethrin than with the pyrethroid beta-cyfluthrin alone. Moreover, for beta-cyfluthrin, Athanassiou et al. [7] found that, on concrete, although knockdown of the exposed adults of *T*. *castaneum* was high after 7 d of exposure, mortality ranged between 33 to 52%. Conversely, knockdown was low for the other two insecticides, especially for chlorfenapyr, probably due to the fact that it is not a neurotoxin. On concrete, knockdown was extremely low for all insecticides, and it is well established that most insecticides are less effective on concrete than on other non-porous surfaces, such as metal or tile surfaces [20–24]. Concrete is porous, so it absorbs a portion of the insecticide, and also alkaline, which is can increase degradation and breakdown of insecticides [11, 21].

Most of the data for the comparison of insecticides that are used in storage facilities are based on longer observation periods (usually >1 d), and there are comparatively less data available for efficacy of insecticides immediately after their application. Chadwick [25] underlined the importance of surface in the initial insecticidal activity and residual efficacy of pyrethroids. Based on our results, concrete negatively affected knockdown, at least during the first hour post-application, regardless of the insecticide tested. We assume that this is probably due to rapid absorption of the water-based insecticide emulsion.

In our tests, we used flour as a food source, in order to reduce control mortality due to starvation. In a recent study, Athanassiou et al. [7] reported that the efficacy of beta-cyfluthrin on concrete for the control of adults of *T*. *castaneum* was lower when adults were provided with food compared to when no food was provided, even though the adults were knocked down. This means that adults that are knocked down may be able to move and gain access to food, resulting in increased recovery from knockdown. In our study, those that were ranked as “3” (with only a minimal movement), may be were able to move to some degree. Consequently, this suggests that the condition of knockdown is dynamic and may change through time not only “upwards” (i.e. individuals that have been ranked initially with “1” to move to “2” or “3” in the next days of exposure) but also “downwards” (i.e. individuals that have been ranked with “3” to move to “2” or “1” in the next days of exposure). In this context, ranking is highly dependent on the time of the observation, as it captures only a short period of knockdown. However, for other beetle species, including *O*. *surinamensis*, the presence of food does not increase survival, which means that the level of knockdown does not change much through time, and immobilization may be more consistent [7]. Similar results have been reported by Vassilakos et al. [11] for surfaces treated with the bacterial insecticide spinetoram.

The presence of food during the post-exposure period is positively correlated with survival [26–27]. Arthur [26] exposed adults of *T*. *castaneum* to chlorfenapyr for short intervals (2–8 h) and found that survival was high when food was present after exposure. This is particularly important with chlorfenapyr, which acts more slowly than other insecticides. Moreover, in “real world” conditions, there are areas (surfaces) that may be only partially treated, under-dosed or even untreated, which means that if there is no knockdown, the insects can move to insecticide-free areas and survive. It remains unclear, however, if the insects that have been exposed for short periods to chlorfenapyr will die even after their removal from the treated substrate, or a continuous contact with the treated surface is needed to obtain high levels of mortality. It is not clear if the absence of knockdown with non-neurotoxic insecticides also leads to increased survival [4, 21, 26]. Additional experimentation is needed with chlorfenapyr, but also with other non-neurotoxic insecticides, to examine if short exposures without knockdown leads to delayed mortality after the removal from the treated substrate. For *T*. *confusum* and *T*. *castaneum*, Arthur [17] found that at exposures <1 d to chlorfenapyr did lead to delayed mortality. For stored product psocids, Guedes et al. [9] reported changes in the mobility patterns of the exposed psocides after they contacted concrete that had been treated with chlorfenapyr. However, this could be due to the fact that psocids are considered very susceptible to chlorfenapyr [28].

Among the insecticides examined here, chlorfenapyr was the only one that was more effective on concrete than on metal, while the reverse was noted for the other three insecticides. This observation stands in accordance with Arthur [17] who found that an EC (Emulsifiable Concentrate) formulation of chlorfenapyr was more effective against *T*. *confusum* and *T*. *castaneum* on concrete than on tile and plywood. While, theoretically, many EC formulations are absorbed on concrete [21, 25], it seems that there are specific interactions between concrete and chlorfenapyr that did not occur in with other insecticides. Arthur [17] suggested that uptake and distribution of chlorfenapyr on concrete may be different than other active ingredients.

Leksey et al. [2] designed the lethality index, which was based on 37 insecticidal treatments, and found that knockdown was generally positively correlated with mortality. The current index differs from Leskey et al. [2] because it gives different levels of knockdown, arranged on a manner so that the weights are normalized (the sum of them equals to 1). The use of an index that gives different knockdown patterns after exposure to insecticides may serve as a clear indicator that connects the “speed of knockdown” with the “speed of kill”. In our modification we divided knockdown in three categories, with each one giving a different “weight” to the overall index value. Consequently, “heavy” knockdown (classified here as “3”) contributed greatly to the increase of the value of the index, despite the fact that mortality was not complete (100%) in the exposure interval tested here (14 d). Nevertheless, there were cases on which insects were knocked down at least during the first 5 d of exposure, but mortality levels were low. For example, on surfaces that had been sprayed with alpha-cypermethrin we noted that the exposed adults, for both species, were rapidly knocked down, but mortality was increased more gradually than for the other insecticides, especially on concrete. This means that for some insecticides, particularly pyrethroids, insects can remain at the knockdown state for a long period. In surface treatments, where insecticides act through contact, if knockdown lasts for long, then survival is likely to occur eventually, either through the degradation of the insecticide from the treated surface, or through detoxification of the active ingredient in the insects’ bodies [1, 3, 7, 21, 25]. According to Georgiou [1], rapid knockdown is not desirable, as it stops the contact of the target individual with the toxic agent, which means that insect can detoxify this agent and survive. Conversely, as noted above, the absence of knockdown may also contribute to increased survival [17, 29]. Sehgal et al. [19] found that recovery of various strains of *R*. *dominica*, *O*. *surinamensis* and *T*. *casteneum* from exposure to concrete surfaces treated with either beta-cyfluthrin or chorpyriphos-methyl + deltamethrin was noticeable, which suggests that these insecticides had no delayed effects. Recovery is probably more likely to occur for some species for which their knockdown patterns are closer to “1”, as compared with other species for which knockdown is closer to “3”. Based on our data but also on previous reports, adults of *T*. *confusum* can be considered as more likely to recover than adults of *O*. *surinamensis* [7, 11, 30]. Leksey et al. [2] found that for several insecticides, mostly pyrethroids but also for the neonicotinoid acetamiprid, the lethality index value was reduced, as recovery from the moribund stage was high. One possible approach to quantitatively examine this phenomenon is to design a “binary” index that assesses all stages from lethality to recovery, by removing the insects after a certain interval and then reclassifying the surviving individuals with the same scaling (from 0 to 4). In this way, the reduction of the value of the index during the removal stage will serve as an indicator of recovery, just like the increase of the value of the index can serve as an indication of mortality.

The index, as designed here, provides a standardized knockdown-to-mortality scaling, with a non-parametric approach. However, this “standardized” approach is based on the weight of specific averaged observations, so it “conceals” possible differences among categories for insecticides that provide different knockdown patterns. In the current study, chlorfenapyr showed no (or very low) knockdown, but, at the same time, mortality was high even at short exposure intervals. In this regard, the majority of the exposed individuals were ranked either as “0” or “4”, indicating alive or dead, while there were very few adults that were ranked with the other three categories during the observation period. By standardizing this, the index averaged the two extreme nominal values of “0” and “4” to a certain level that was often <100, despite the fact that that there was little knockdown. These values for chlorfenapyr were sufficiently different than similar values for other insecticides, such as alpha-cypermethrin, for which knockdown was rapid, but mortality was generally low, so the index values were mostly based on the weighted values of “1”, “2” or “3”. This makes the index less descriptive regarding the dynamic changes among knockdown categories over time.

In the present study, we made an attempt to quantify knockdown of two major stored product insect species, by assessing several commonly used insecticides with different mode of action. Our results show that all four insecticides were effective in controlling *T*. *confusum* and *O*. *surinamensis* on metal and concrete surfaces. Apparently, recoding the values for the calculation of the index is depended on the time of observation, and the three out of the five ranks of the index (1–3) are rather subjective, as they depend on the person that collects the observations during the experimental part. Still, the current index provides valuable information on the evaluation of knockdown as a scaling factor that is related with both mortality and recovery. This index, named here as Standardized Lethality Index, can be further expanded to include recovery patterns and also prediction estimates that connect knockdown with mortality.

## Supporting Information

S1 Dataset Knockdown data set. (XLSX)

S2 Dataset Lethality index data set. (XLSX)
